# Antitumor Effect of *n*-Butylidenephthalide Encapsulated on B16/F10 Melanoma Cells In Vitro with a Polycationic Liposome Containing PEI and Polyethylene Glycol Complex

**DOI:** 10.3390/molecules23123224

**Published:** 2018-12-06

**Authors:** Hong-Wei Gao, Kai-Fu Chang, Xiao-Fan Huang, Yu-Ling Lin, Jun-Cheng Weng, Kuang-Wen Liao, Nu-Man Tsai

**Affiliations:** 1 Department of Pathology, Tri-Service General Hospital, National Defense Medical Center, Taipei 12742, Taiwan; doc31796@gmail.com; 2 Institute of Medicine of Chung Shan Medical University, Taichung 9012, Taiwan; kfchang1015@gmail.com (K.-F.C.); s9870509@gmail.com (X.-F.H.); 3 Department of Medical Laboratory and Biotechnology, Chung Shan Medical University, Taichung 9012, Taiwan; 4 Department of Biological Science and Technology, National Chiao Tung University, Hsinchu 51329, Taiwan; lyring@pchome.com.tw (Y.-L.L.); liaonms@pchome.com.tw (K.-W.L.); 5 Agricultural Biotechnology Research Center, Academia Sinica, Taipei 43433, Taiwan; 6 Department of Medical Imaging and Radiological Sciences, Chang Gung University, Taoyuan 24334, Taiwan; jcweng@gmail.com; 7 Institute of Molecular Medicine and Bioengineering, National Chiao Tung University, Hsinchu 51329, Taiwan; 8 Clinical Laboratory, Chung Shan Medical University Hospital, Taichung 4720, Taiwan

**Keywords:** melanoma, polycationic liposome containing PEI and polyethylene glycol complex (LPPC), *n*-Butylidenephthalide

## Abstract

Advanced melanoma can metastasize to distal organs from the skin and yield an aggressive disease and poor prognosis even after treatment with chemotherapeutic agents. The compound *n*-Butylidenephthalide (BP) is isolated from *Angelica sinensis*, which is used to treat anemia and gynecological dysfunction in traditional Chinese medicine. Studies have indicated that BP can inhibit cancers, including brain, lung, prostate, liver, and colon cancers. However, because BP is a natural hydrophobic compound, it is quickly metabolized by the liver within 24 h, and thus has limited potential for development in cancer therapy. This study investigated the anticancer mechanisms of BP through encapsulation with a novel polycationic liposome containing polyethylenimine (PEI) and polyethylene glycol complex (LPPC) in melanoma cells. The results demonstrated that BP/LPPC had higher cytotoxicity than BP alone and induced cell cycle arrest at the G_0_/G_1_ phase in B16/F10 melanoma cells. The BP/LPPC-treated cell indicated an increase in subG_1_ percentage and TUNEL positive apoptotic morphology through induction of extrinsic and intrinsic apoptosis pathways. The combination of BP and LPPC and clinical drug 5-Fluorouracil had a greater synergistic inhibition effect than did a single drug. Moreover, LPPC encapsulation improved the uptake of BP values through enhancement of cell endocytosis and maintained BP cytotoxicity activity within 24 h. In conclusion, BP/LPPC can inhibit growth of melanoma cells and induce cell arrest and apoptosis, indicating that BP/LPPC has great potential for development of melanoma therapy agents.

## 1. Introduction

The compound *n*-Butylidenephthalide (BP) can be isolated from *Angelica sinensis*—also called dong quai—which is primarily grown in Asia and has major applications in the treatment of anemia and gynecological dysfunction in traditional Chinese medicine [1,2]. Moreover, BP is a natural liposoluble compound with a low molecular weight (188.226 g/mol) that possesses multiple pharmacological activities, such as reducing injury and fibrosis in the liver and neuroprotective activity [3,4,5]. Studies have revealed that BP exerts cytotoxic or inhibitory effects on various cancers, including brain, lung, colon, prostate, and liver cancers [6,7,8,9,10,11,12]. However, no anti-melanoma effect was recorded until a study of BP on melanoma in vitro. 

Although BP has anticancer effects, the clinical application for BP is limited by its properties, namely poor dissolution, forming of dimers, easy hydration, and oxidization influencing bioavailability [13]. To overcome these limitations and create an effective therapeutic agent, drug carriers have been developed. For example, a polycationic liposome complex containing PEI and PEG (Lipo-PEG-PEI complex; LPPC) is a drug carrier comprising DOPC (1,2-Dioleoyl-sn-glycero-3-phosphocholine), DLPC (1,2-dilauroyl-sn-glycero-3-phosphocholine), PEG (Polyethylene glycol), and PEI (polyethylenimine), and with properties including a positive charge with 40 mV, protein capture, drug entrapment into lipophilic or hydrophilic structures, and components that can be metabolized by the liver. Moreover, curcumin-loaded LPPC can improve the cytotoxic activity of curcumin, resulting in cancer cell death [14,15,16,17]. Here, we wondered whether the modification of a drug delivery system with LPPC may overcome the drawbacks of BP and reduce the required dosage to enhance drug efficacy. 

Studies have indicated that nanoparticles with positive charges provide a method for entrapping drugs into cells through means such as the phagocytosis, clathrin-mediated, caveolae-mediated, clathrin-independent, caveolae-independent, and macropinocytosis pathways [18,19]. However, the antimelanoma mechanism of an LPPC-encapsulated BP system has not been elucidated. The present study demonstrated that the anticancer effect of BP in melanoma cells is mediated through cell cycle arrest and apoptosis mechanisms. In addition, this study established a novel BP nanoparticle system to overcome poor water solubility and ameliorate the anticancer effect for comparison with free BP.

## 2. Results

### 2.1. Cytotoxicity of BP/LPPC in Tumor and Control Cells

**Figure 1:**
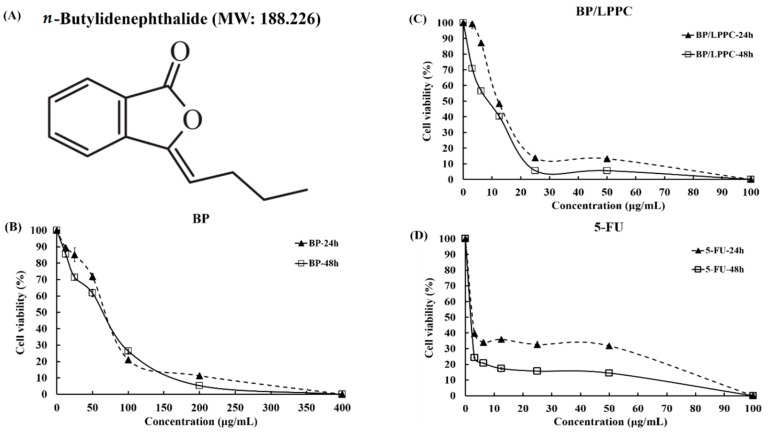
The inhibition curve of *n*-Butylidenephthalide (BP)/polyethylene glycol complex (LPPC) on B16/F10 melanoma cells. (**A**) The chemical structure and molecular weight of *n*-Butylidenephthalide (BP). B16/F10 cells were plated in 96-well culture plates and treated with (**B**) BP (0–400 µg/mL), (**C**) BP/LPPC (0–100 µg/mL), and (**D**) 5-Fluorouracil (5-FU) (0–100 µg/mL) with serial dilution for 24 and 48 h. The cell viability was measured using MTT assay, and calculated by the following: cell viability (%) = (O.D. value of treatment group/O.D. value of control group) × 100%. The results showed mean ± SD.

The cells were treated with BP, BP/LPPC, and 5-Fluorouracil (5-FU) for 24 and 48 h, and cell viability was measured using MTT. The results revealed that BP and BP/LPPC inhibited melanoma cell growth in a dosage-dependent manner, as shown in Figure 1B–D. The cytotoxicity of BP/LPPC (IC_50_ = 12.24 ± 1.22 to 15.28 ± 1.94 µg/mL) was higher than those of BP (56.49 ± 0.51 to 71.47 ± 1.97 µg/mL) and BP/Lipo (IC_50_ = 83.78 ± 2.96 to 108.89 ± 2.34 µg/mL) in the tumor cells but lower in the control cells (IC_50_ = 23.36 ± 0.27 to 50.4 ± 3.6 µg/mL) at 24 h, as shown in Table 1. However, 5-FU indicated high cytotoxicity in the tumor cells and control cells (IC_50_ = 2.66 ± 1.46 to 9.20 ± 4.66 µg/mL).

### 2.2. BP/LPPC-Induced Cell Cycle Arrest (G_0_/G_1_) in B16/F10 Melanoma Cells

**Figure 2:**
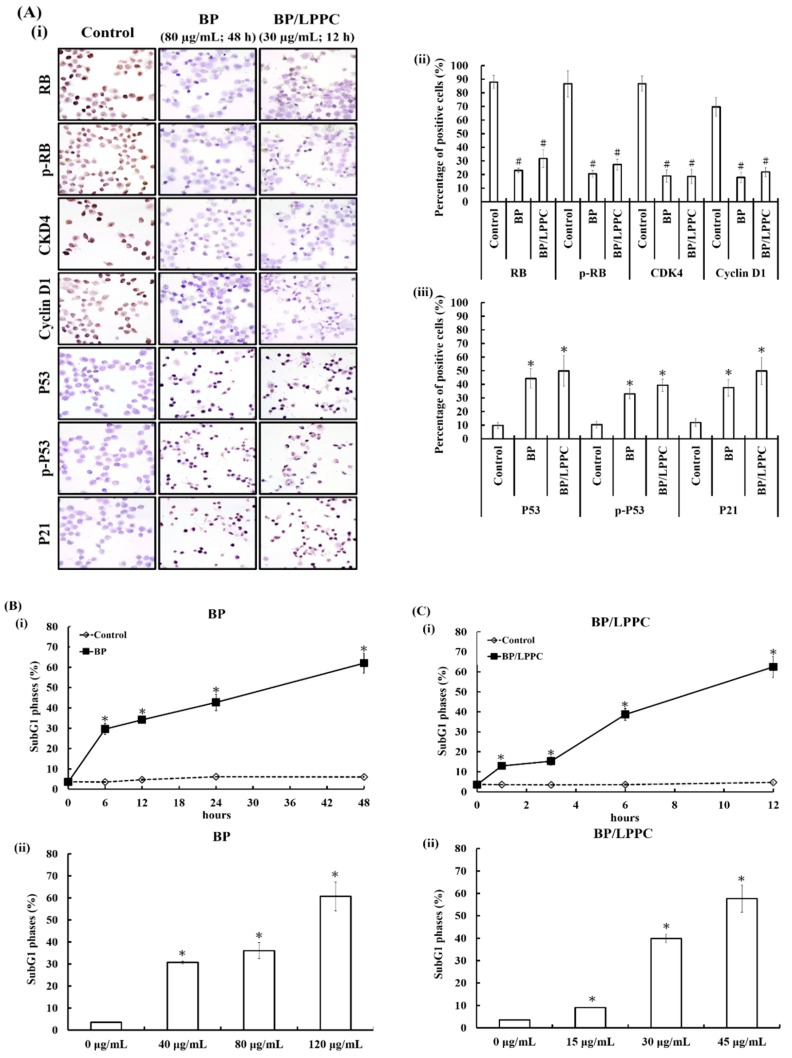
BP/LPPC downregulated cell cycle related protein expression and increased percentage of SubG1 on melanoma cells. (**A**) Cells were treated with BP (80 µg/mL for 6–12 h) and BP/LPPC (60 µg/mL for 24–48 h) and detected protein expression of RB, p-RB, CDK4, Cyclin D1, P53, p-P53 and P21 using immunocytochemistry staining. ^#^ *p* < 0.05 versus control with significant decrease. * *p* < 0.05 versus control with significant increase. Cells were treated with (**B**) BP and (**C**) BP/LPPC with time course and dosage, and analyzed percentage of subG_1_ phase using flow cytometry analysis with propidium iodide staining. Data represents the mean ± SD; * *p* < 0.05 versus control.

The cell cycle distribution of the B16/F10 cells was treated with BP (0–120 µg/mL; 0–48 h) or BP/LPPC (0–45 µg/mL; 0–12 h), resulting in cell cycle arrest at the G_0_/G_1_ phase in a time course and dosage-dependent manner (*p* < 0.05), as shown in Table 2. Comparison of conditions of BP and BP/LPPC induced 66% cell cycle arresting at the G_0_/G_1_ phase, BP needed 80 µg/mL treated for 24 h and BP/LPPC needed only 30 µg/mL treated for 6 h. From this result, BP/LPPC induced cell cycle arresting more efficiently than BP in low dose or short time conditions. BP and BP/LPPC treatment reduced the proportion of cell cycles in the S and G_2_/M phase. Moreover, BP- and BP/LPPC-treated cells showed decreased protein expression of RB, p-RB, CDK4, and cyclin D1 and increased protein expression of P53, p-P53, and P21, which led cell cycle arrest at the G_0_/G_1_ phase, as shown in Figure 2A(i) to (iii). After BP and BP/LPPC treatment for time course and dosage, the cells were collected and analyzed for the subG1 phase using flow cytometry. The results showed that the percentage of the subG_1_ phase had increased after BP or BP/LPPC treatment in time course and dosage-dependent manners, as shown in Figure 2B,C.

### 2.3. Morphological Evaluation and Mechanism of BP/LPPC-Induced Apoptosis

**Figure 3:**
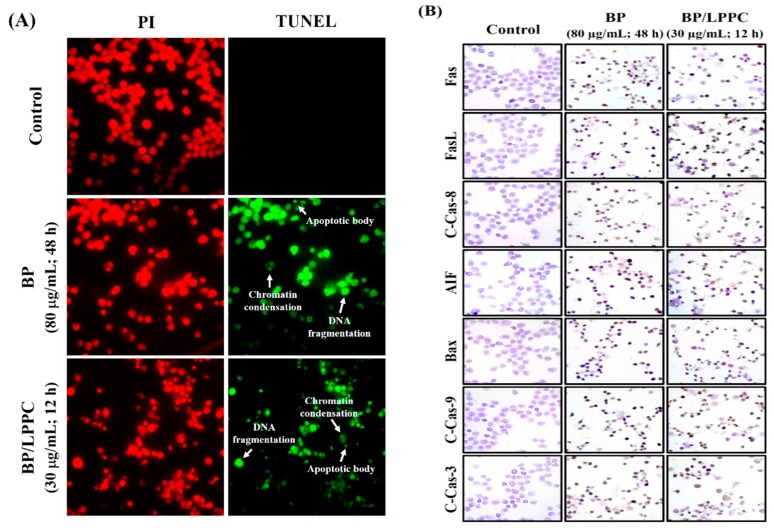

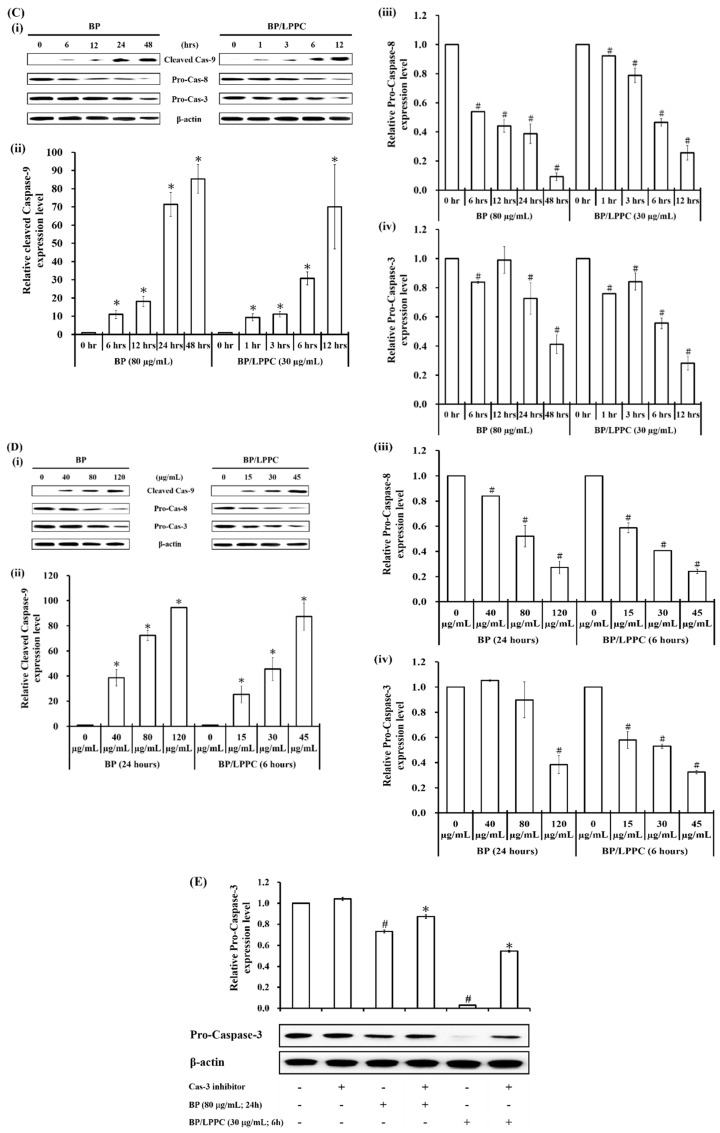
BP/LPPC-induced apoptosis morphology and associated protein expression on B16/F10 cells. (**A**) BP or BP/LPPC-treated cell showed TUNEL positive results and apoptotic cell morphology, including chromatin condensation, DNA fragmentation, and apoptotic bodies. (**B**) BP or BP/LPPC-induced extrinsic (Fas, FasL, and Cleaved-Caspase-8) and intrinsic (Bax, AIF, and Cleaved-Caspase-9) apoptotic pathways and downstream Cleaved-Caspase-3 activation by immunocytochemistry staining. (**C**) The protein expression of Cleaved-Caspase-9, Pro-Caspase-8, and Pro-Caspase-3 were analyzed by western blotting. ^#^ *p* < 0.05 versus control with significant decrease. * *p* < 0.05 versus control with significant increase. (**D**) To confirm BP/LPPC-induced cell death through apoptosis pathway, B16/F10 pretreated with Caspase-3 inhibitor (1 μM Z-DEVD-FMK) before BP/LPPC treatment. Cleaved-Caspase-3 was detected using western blotting. The columns showed mean ± SD. ^#^ *p* < 0.05 versus control with significant decrease. * *p* < 0.05 combination group versus BP or BP/LPPC only with significant increase. (**E**) The activation of Caspase-3 was blocked when B16/F10 cells were pretreated with an inhibitor. ^#^ *p* < 0.05 combination group versus BP or BP/LPPC only with significant decrease. * *p* < 0.05 combination group versus BP or BP/LPPC only with significant increase.

To investigate drug-induced cell death through the apoptosis pathway, the cells were stained using a TUNEL assay after BP or BP/LPPC treatment. The BP- and BP/LPPC-treated cells indicated a positive TUNEL result and apoptotic morphology, including chromatin condensation, DNA fragmentation, and presence of apoptotic bodies, as shown in Figure 3A. The immunocytochemistry staining results indicated that BP and BP/LPPC activated extrinsic (Fas, FasL and Claved-Cas-8) and intrinsic (Bax, AIF, and Cleaved-Cas-9) apoptosis pathways and triggered downstream Cleaved-Cas-3 activity, as shown in Figure 3B. Moreover, Caspase-3, -8, and -9 were activated after BP and BP/LPPC treatment in time course and dosage-dependent manners using western blotting analysis, as shown in Figure 3C,D. To determine whether caspase cascade was activated by BP or BP/LPPC, the cells were pretreated with Caspase-3 inhibitor before BP and BP/LPPC treatment. The results revealed that activation of Caspase-3 was blocked when the cells were pretreated with an inhibitor, as shown in Figure 3E. These results demonstrated that BP- and BP/LPPC-induced cell death through activation of extrinsic and intrinsic apoptosis pathways.

### 2.4. Combination of BP/LPPC and 5-FU had a Synergistic Effect

**Figure 4:**
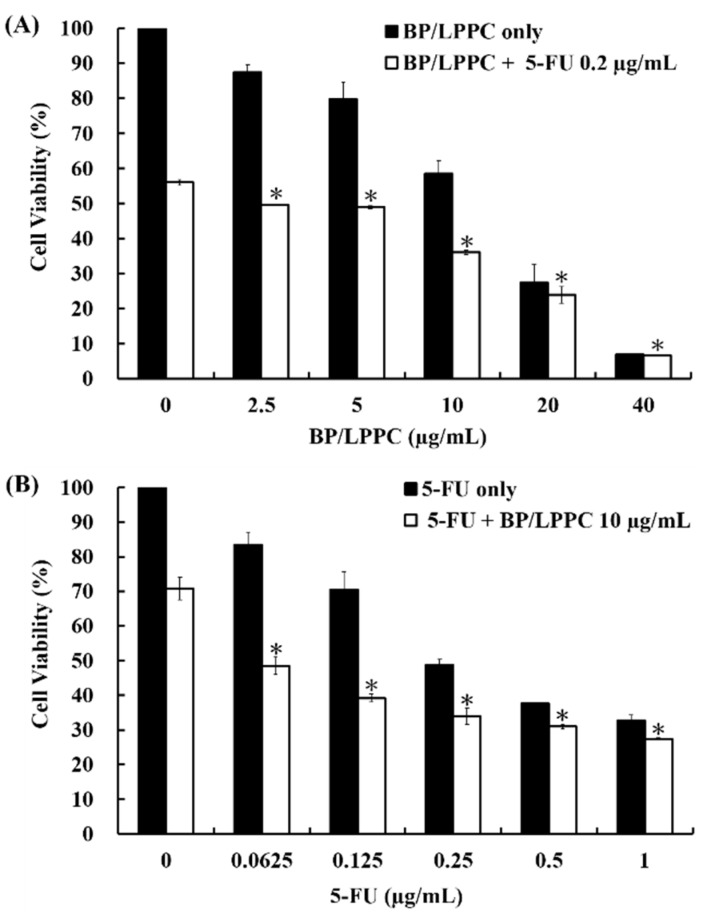
Combination of BP/LPPC and 5-FU revealed a synergistic effect on melanoma cells. The cells were treated with (**A**) BP/LPPC (0–40 µg/mL) combined with 5-FU (0.2 µg/mL); (**B**) 5-FU (0–1 µg/mL) combined with BP/LPPC (10 µg/mL). The cell viability was measured by MTT assay. * *p* < 0.05 versus control dose (0 µg/mL) in combination group.

The B16/F10 cells were treated with a serial concentration of BP/LPPC combined with 5-FU (0.2 µg/mL) or a serial concentration of 5-FU combined with BP/LPPC (10 µg/mL) for 48 h. The cell viability of the combination group (56.01 ± 0.78% to 6.63 ± 0.16% in BP/LPPC combined with 0.2 µg/mL 5-FU; 70.81 ± 3.18% to 27.34 ± 0.31% in 5-FU combined with 10 µg/mL BP/LPPC) was lower than that of the single drug group (100 ± 1.39% to 6.88 ± 0.19% in BP/LPPC only; 100 ± 3.14% to 32.73 ± 1.58% in 5-FU only) in a dosage-dependent manner, with a combination index (CI) of 0.42, as shown in Figure 4A,B. Moreover, the cell viability of the combination group significantly decreased compared with the BP/LPPC or 5-FU drug only-treated group (*p* < 0.05). These results suggested that BP/LPPC combined with 5-FU had a synergistic effect on the cytotoxicity of melanoma cells.

### 2.5. Protection Effect of BP Encapsulated with LPPC

**Figure 5:**
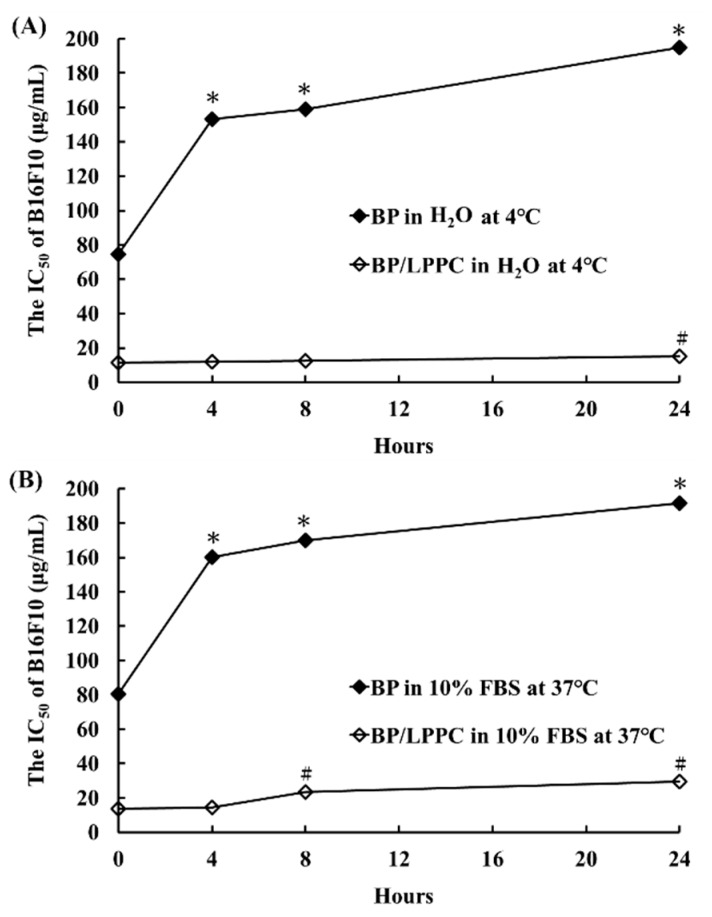
LPPC encapsulation stabilized and maintained BP activity for cytotoxicity of melanoma cells. The LPPC non-capsulated BP (BP group) and capsulated BP (BP/LPPC group) were stored in (**A**) ultra pure and sterile water (H_2_O) at 4 °C or (**B**) 10% fetal bovine serum (FBS) in phosphate-buffered saline (PBS) at 37 °C environment for 0, 4, 8, and 24 h and calculated cytotoxicity (IC_50_) of BP using MTT assay. * *p* < 0.05 versus control dose (0 µg/mL) in BP group. **^#^** *p* < 0.05 versus control dose (0 µg/mL) in BP/LPPC group.

To investigate whether LPPC encapsulation protected and maintained BP′s cytotoxic activity, BP was encapsulated with and without LPPC and stored in ultra pure and sterile water (H_2_O) at 4 °C or in a protein-rich environment at 37 °C and incubated for 0, 4, 8, and 24 h. The activity of BP was evaluated based on its cytotoxicity in B16/F10 cells. BP/LPPC exhibited a higher cytotoxicity (IC_50_ = 15.21 ± 0.04 µg/mL in H_2_O; 29.55 ± 0.78 µg/mL in 10% fetal bovine serum (FBS) at 24 h) than did BP without LPPC encapsulation (IC_50_ = 194.83 ± 4.11 µg/mL in H_2_O; 191.57 ± 2.30 µg/mL in 10% FBS at 24 h) in all environments, as shown in Figure 5A,B, thereby indicating that LPPC encapsulation protected and maintained BP activity to enhance the cytotoxicity of BP in melanoma cells.

### 2.6. LPPC with Positive Charge Triggered Cell Uptake of BP through the Endocytosis Pathway

**Figure 6:**
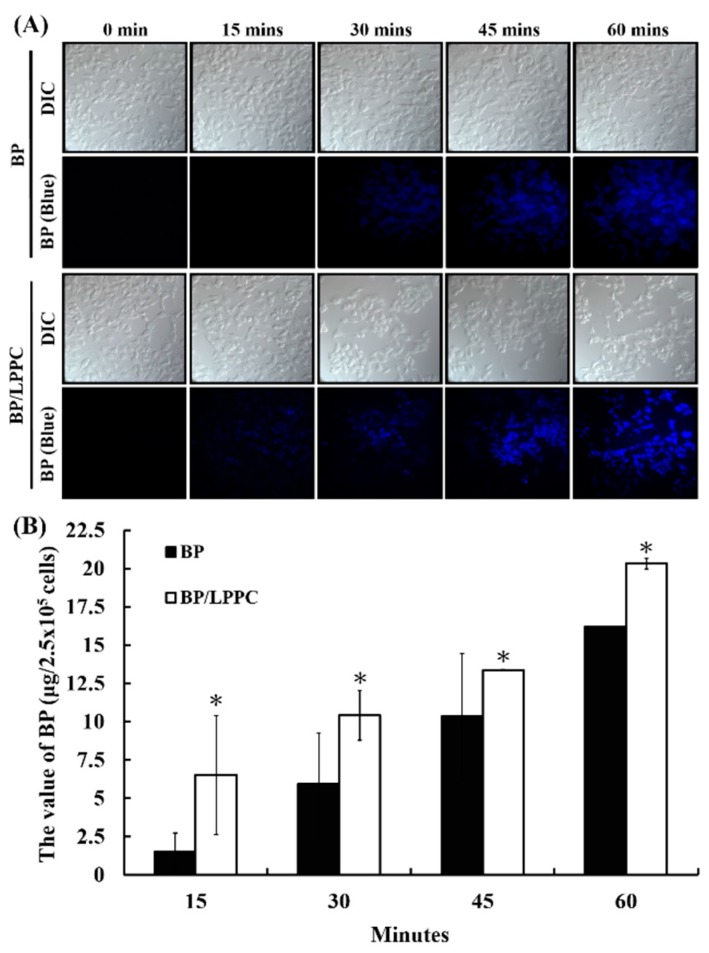
LPPC encapsulation enhanced cell uptake of BP through induction of endocytosis. (**A**,**B**) The cells were incubated with BP (50 µg/mL) or BP/LPPC (50 µg/mL) for 0, 15, 30, 45, and 60 min, and then extracted for BP value analysis using a fluorescence spectrophotometer. The fluorescence of BP was blue. * *p* < 0.05 versus BP group.

After the cells had been incubated with BP or BP/LPPC, BP (blue fluorescence) in the cells was observed at 15 min in the BP/LPPC group and 30 min in the BP group, as shown in Figure 6A. In the quantitative analysis, the BP value of cell uptake in the BP/LPPC group (6.52 ± 3.89 to 20.32 ± 0.36 µg per 2.5 × 10^5^ cells) was higher than that in the BP only group (1.51 ± 1.22 to 16.19 ± 0.01 µg per 2.5 × 10^5^ cells) for melanoma cells, as shown in Figure 6B. These data indicated that BP/LPPC penetrated the cells more quickly than did BP. Therefore, the pathway of cell uptake induced by LPPC encapsulation was investigated next. Through use of different endocytosis inhibitors, the BP value of cell uptake was decreased after pretreatment with all endocytosis inhibitors, namely AHH (2.53 ± 0.46 to 8.38 ± 0.25 per 2.5 × 10^5^ cells; micropinocytosis pathway), FIII (5.69 ± 1.28 to 12.77 ± 0.06 per 2.5 × 10^5^ cells; caveolae-mediated endocytosis pathway), and CPZ (2.42 ± 0.25 to 12.42 ± 0.52 per 2.5 × 10^5^ cells; clathrin-mediated endocytosis pathway), as shown in Table 3. This suggested that BP/LPPC with a positive charge induced varying degrees of endocytosis to enhance cell uptake of BP in melanoma cells.

## 3. Materials and Methods

### 3.1. Preparation of BP/LPPC

*n*-Butylidenephthalide (BP), (E) + (Z), 95% was purchased from Alfa Aesar, Thermo Fisher Scientific (Waltham, MA, USA), and its chemical structure was described in a previous study [1], as shown in Figure 1A. The LPPC used in this study was provided by National Chiao Tung University, Hsinchu, Taiwan. A total of 100 μL of LPPC was added to 900 μL of ultra pure and sterile water (H_2_O) and the mixture was centrifuged (Microcentrifuges, Force 1624, Select BioProducts, Edison, NJ, USA) at 9000 rpm for 5 min to remove the supernatant. The pellet was resuspended in 100 μL of H_2_O and strongly vortexed after addition of 20 μL of 1 M BP mixed with 10 μL of dimethylsulfoxide (DMSO). After incubation for 30 min at room temperature, the unencapsulated BP was removed and the pellet was resuspended in a solution containing 750 μL of H_2_O and 750 μL of PEG-1500 solution (100 mg/mL, Acros Organics, Morris Plains, NJ, USA) for 30 min at room temperature. The pellet (BP/LPPC) was stored at 4 °C and treated for cells for 1 day. The BP value of the LPPC encapsulation equaled the total BP value minus the BP value of the LPPC unencapsulated in the supernatant. The BP value in the supernatant was measured using a fluorescence spectrophotometer at 350 nm (Hitachi F4500, Hitachi Instruments Inc., Tokyo, Japan). The BP/LPPC were prepared according to a previously described manufacturing process and analyzed the characteristics, including particle sizes (200 nm to 280 nm), average zeta-potential (~38 mV), drug release, and encapsulation capacity [13]. 

### 3.2. Cell Culture and Reagent

The cancer cells used were B16/F10 (mouse melanoma cells) and K-Blab (mouse fibroblast sarcoma cells). The control cells were Blab/3T3 (mouse fibroblast), MDCK (canis kidney epithelial cells), and SVEC (mouse endothelial cells), all of which were purchased from the Food Industry Research and Development Institute (Hsinchu, Taiwan). The medium used for all cells was Dulbecco’s Modified Eagle’s Medium, containing 10% heat inactivated fetal bovine serum (FBS; Gibco BRL, Gaithersburg, MD, USA), HEPES (10 mM; Gibco), pyruvate (1 mM; Gibco), and P/S (100 U/mL penicillin and 100 μg/mL streptomycin; Gibco). Cells were cultured and incubated in a growth medium in a 37 °C humidified atmosphere with 5% CO_2_. BP (Alfa Aesar, Haverhill, MA, USA) and 5-Fluorouracil (5-FU; Sigma, Setagaya, Tokyo, Japan) were dissolved in DMSO and stored at 4 °C or −20 °C in each in vitro experiment.

### 3.3. BP/LPPC-Induced Cytotoxicity

The cytotoxicity of the drugs was estimated based on cell viability and detected by modified 3-(4,5-dimethylthiazol-2-yl)-2,5-diphenyltetrazolium bromide assay (MTT). The cells were plated on 96-well culture plates (5 × 10^3^ per well) and incubated overnight, and then treated with a serial concentration of BP (0–400 μg/mL) or BP/LPPC (0–100 μg/mL) dissolved in a medium for 24 or 48 h, respectively. After removal of the medium, the cells of each concentration were reacted with 100 μL of MTT solution (400 μg/mL, Sigma) for 6–8 h. The MTT solution was replaced with 50 μL of DMSO to dissolve formazan crystals and detect optical density (O.D.) values using a microplate reader (Molecular Device, Spec384, San Jose, CA, USA) at 550 nm. The viability of the cells in the medium only was used as a control and regarded as 100% viable.

### 3.4. Analysis of Cell Cycle Arrest Induced by BP/LPPC

The B16/F10 cells were plated in a 10 cm^2^ dish (2 × 10^6^ per dish) overnight and treated with 80 μg/mL of BP (for 0, 6, 12, 24, and 48 h) and 30 μg/mL of BP/LPPC (for 0, 1, 3, 6, and 12 h) for time course analysis, or various concentrations of BP (40, 80, and 120 μg/mL) for 24 h and BP/LPPC (15, 30, and 45 μg/mL) for 6 h for dosage analysis. After incubation, the cells were harvested, washed, and resuspended with 1000 μL of phosphate-buffered saline (PBS) containing propidium iodide (PI) (40 μg/mL, Sigma) and RNase (100 μg/mL, Sigma), and incubated at 4 °C overnight. The FL2 intensity of the cell cycle was analyzed using FACScan (Becton Dickinson, Franklin Lakes, NJ, USA) and Kaluza Flow Cytometry Analysis Software (Version 1.2, Beckman Coulter, Brea, CA, USA).

### 3.5. TUNEL Assay

Drug-induced apoptosis was determined using an In Situ Cell Death Detection Kit, POD (Roche, Mannheim, Germany). The B16/F10 cells were seeded on 10 cm^2^ dishes overnight and incubated with 80 μg/mL of BP for 24 h and 30 μg/mL of BP/LPPC for 6 h. The cells were harvested, washed, and fixed with 10% formaldehyde, and dried on silane-coated glass slides (Matsunami, Tokyo, Japan). After being washed, the cells on the slides were treated with 3% H_2_O_2_ to decrease the activity of endogenous peroxidase, and then incubated with cold 0.1% Triton X-100 in 0.1% sodium citrate to increase the permeability of the cells on ice. The cells were washed with PBS, incubated with the TUNEL reaction mixture for 2 h at 37 °C, and then counterstained with PI (10 μg/mL, Sigma). The morphology of cell apoptosis (apoptotic body, DNA fragmentation, and DNA condensation) was observed using a fluorescence microscope (ZEISS AXioskop2, Carl Zeiss, Munich, Germany) at a magnification of ×400.

### 3.6. Immunocytochemistry

BP- and BP/LPPC-treated cells were collected, fixed with 4% paraformaldehyde, and incubated with Triton X-100 to rupture the cell membranes. A sample slide was blocked with 10% BSA for 30 min at 37 °C and incubated with antibodies, namely anti-P53, anti-p-P53, anti-RB, anti-p-RB, P21, CDK4, Cyclin D1, anti-Fas, anti-Fas-L, anti-Cleaved-Caspase-8, anti-Bax, anti-AIF, anti-Cleaved-Caspase-9, and anti-Cleaved-Caspase-3 (1/200 dilution; Santa Cruz Biotechnology, Inc., Dallas, TX, USA), overnight at 4 °C. The cells were incubated with an appropriate horseradish peroxidase-conjugated antimouse, antirabbit, and antigoat IgG secondary antibody (1/1500 dilution; Santa Cruz Biotechnology, Dallas, TX, USA) for 1 h at room temperature, and then visualized with DAB substrate solution and counterstained with hematoxylin. The slides were observed and photographed under a light microscope at a magnification of ×400 (ZEISS AXioskop2).

### 3.7. Western Blotting

The B16/F10 cells were incubated in a 10 cm^2^ dish (2 × 10^6^ cells per dish) overnight and treated with BP (0, 40, 80, and 120 μg/mL) for 0–48 h or BP/LPPC (0, 15, 30, and 45 μg/mL) for 0–12 h. The cells were collected and washed and then lysis was conducted with a radioimmunoprecipitation assay (RIPA) buffer mixture solution containing 1 RIPA buffer (Bio Basic Inc., Markham Canada) and 1 protease inhibitor (Bio Basic Inc., Canada) on ice for 30 min. The concentration of proteins was detected using a bicinchoninic acid protein assay kit (Pierce, Rockford, IL, USA). The proteins (20 μg/sample) were separated using 10–12% sodium dodecyl sulfate polyacrylamide gel electrophoresis, transferred to polyvinylidene difluoride membranes (FluoroTrans, PALL, Dreieich, Germany), and blocked with 5% skin milk for 30 min at 25 °C. The membranes were incubated for primary antibodies in a Tris-buffered saline buffer containing 1% BSA overnight at 4 °C. The first antibodies were anti-Caspase 3, anti-Caspase 8, anti-Caspase 9, and anti-β-actin (1/200 dilution; Santa Cruz). The membranes were incubated with horseradish peroxidase-conjugated antimouse or antirabbit secondary antibodies (1/1500 dilution; Santa Cruz) at 25 °C for 2 h and visualized for enhanced chemiluminescence (T-Pro Biotechnology, Taipei, Taiwan). The chemiluminescence on the membranes was detected by a chemiluminescence and fluorescence imaging analyzer (GE LAS-4000, GE Healthcare Life Sciences, NJ, USA). The protein expression indices were calculated as follows: [intensity (sample)/β-actin intensity (sample)]/[intensity (control)/β-actin intensity (control)].

### 3.8. Inhibition Caspase-3 Activity Assay

The B16/F10 cells were incubated on 6-well culture plates (5 × 10^5^ cells per well) overnight, and then the medium of each well was replaced with a Caspase-3 inhibitor (1 μM Z-DEVD-FMK, G-Biosciences, Louis, MO, USA) and incubated for 2 h. After removal of the medium, the cells were treated with BP (80 μg/mL) for 24 h or BP/LPPC (30 μg/mL) for 6 h. The protein expression level of Caspase-3 was detected using western blotting.

### 3.9. Synergistic Effects of BP/LPPC Combined with 5-FU

The B16/F10 cells were seeded on 96-well culture plates (5 × 10^3^ cells per well) overnight and treated with (1) a serial concentration of BP/LPPC (0, 2.5, 5, 10, 20, and 40 μg/mL) combined with 0.2 μg/mL of 5-FU for 48 h, and (2) a serial concentration of 5-FU (0, 0.0625, 0.125, 0.25, 0.5, and 1 μg/mL) combined with 10 μg/mL of BP/LPPC for 48 h. The cell viability of the treated cells was measured using MTT. The combination index (CI) value was calculated as follows: [IC_50_ of (drug A + B)/IC_50_ of (drug A)] + [IC_50_ of (drug A + B)/IC_50_ of (drug B)]. The definition of the CI value indicated synergism (CI < 1), an additive effect (CI = 1), and antagonism (CI > 1). 

### 3.10. Protection of BP Activity through LPPC Encapsulation

To determine the protection of BP activity through LPPC encapsulation, BP and BP/LPPC were resolved in 200 μL of ultra pure and sterile water (H_2_O) or a protein-rich environment solution (10% FBS in PBS), with a final BP concentration of 3 mg/mL in each group; the cells were then stored at 4 °C or 37 °C for 0, 4, 8, or 24 h. After incubation, the cells were treated with incubated BP or BP/LPPC and IC_50_ was calculated using MTT. The protective effect on BP activity was evaluated through the cytotoxicity of BP in the tumor cells.

### 3.11. Cell Uptake of BP/LPPC in Qualitative and Quantitative Analysis

To analyze the cell uptake of BP, the cells were seeded on 15 mm microscope cover glasses (Assistent, Glaswarenfabrik Karl Hecht GmbH & Co KG, Sondheim, Germany) in a 3.5 cm^2^ dish (5 × 10^5^ cells per dish) and incubated overnight. The medium in each dish was replaced with a medium containing 50 μg/mL of BP or BP/LPPC and incubated for 0, 15, 30, 45, or 60 min. After incubation, the cover glasses were removed, washed, and fixed with 10% neutral formalin. The cell uptake of BP was observed through blue fluorescence on an upright fluorescence microscope (ZEISS AXioskop2) at a magnification of ×400. The bright-field was observed under differential interference contrast at a magnification of ×400.

For quantitative analysis of the cell uptake of BP, the cells (2.5 × 10^5^ per well) were incubated in each well of the 24-well culture plate overnight, and the mediums were replaced with 50 μg/mL of BP or BP/LPPC and incubated for 0, 15, 30, 45, and 60 min. Finally, the BP values of the cells were extracted with phenol-chloroform and calculated using a fluorescence spectrophotometer (HITACHI F-4500) at 350 nm [13].

To investigate the endocytosis pathway induced by LPPC encapsulation, the B16/F10 cells were plated on 24-well culture plates (2.5 × 10^5^ per well) and incubated overnight. To remove the medium, 300 μL of medium was added to each well, which contained endocytosis inhibitors of amiloride hydrochloride hydrate (AHH; 13.31 μg/mL, Sigma), Filipin III (FIII; 1 μg/mL, Sigma), or chlorpromazine hydrochloride (CPZ, 10 μg/mL, Sigma) for 1 h incubation. The medium in the well was replaced with 50 μg/mL BP or BP/LPPC and treated for 0, 15, 30, 45, and 60 min. The treated cells were harvested, the BP was extracted with phenol-chloroform, and the BP value in the cells was calculated using fluorescence spectroscopy at 350 nm.

### 3.12. Statistics

In this paper, all results are presented as mean ± standard deviation. The statistical analysis utilized Student’s *t* test to define statistical significance, which was defined as *p* < 0.05.

## 4. Discussion

As several studies have indicated the anticancer activity of BP, our study was the first to demonstrate the anti-melanoma effects of BP with dose-dependent reduction of cell viability at indicated time intervals. Furthermore, BP also exhibited a better inhibitory drug concentration on K-balb, which is mouse fibroblast sarcoma. After that, the effects of BP on different types of cells was examined and the results revealed that the cytotoxic activity of BP toward control cells was lower than tumor cells such as fibroblasts, endothelial cells, and kidney epithelial cells; consequently, the property of BP was selective between tumor and control cells. It is noted that the tumor microenvironment is full of stromal cells, and among them, fibroblasts are the main cell types. For melanoma treatment, the tumor microenvironment affected the therapeutic efficacy due to immunosuppression that was contributed by the tumor associated fibroblasts [20,21,22]. Our results revealed that BP inhibited not only fibroblast sarcoma (K-balb) but also normal fibroblasts (Balb/3T3) in vitro, suggesting BP might have a possibility in targeting fibroblast cells to modulate the tumor microenvironments contributing to a clinical response.

Moreover, another strategy for melanoma treatment has described that lipid nanoparticles provide advantages as a drug delivery system, such as physical stability, controlled release, and good skin permeation. A previous study has found that the LPPC delivery system offered better skin penetration and drug accumulation to improve the therapeutic efficacy in breast cancer [16]. After LPPC carrier encapsulation, LPPC was able to maintain the antimelanoma activity of BP and enhance the tumor cytotoxicity of melanoma cells. Moreover, LPPC encapsulation reinforced the inhibitory effects on both fibroblast sarcoma (K-balb) but also normal fibroblasts (Balb/3T3), indicating LPPC encapsulated BP could improve the anti-fibroblast activity, which ranged from 3.35 to 3.7-fold.

To further investigate the anti-melanoma mechanisms of BP and BP/LLPC, the results of our study indicated that BP and BP/LPPC induced cell cycle arrest by mediated cell cycle regulators’ protein expressions. The data revealed that BP suppressed the protein expression of total RB and phosphorylated RB and downstream CDK4 as well as cyclin D1, contributing to cell cycle arrest at G_0_/G_1_ phase. Additionally, BP also activated phosphorylated p-P53 and P21 protein expressions that both led to cell cycle progression blocking. Meanwhile, after BP/LPPC or BP treatment, BP as well as BP/LPPC was observed to induce cell death via cell apoptosis and BP was able to regulate the extrinsic and intrinsic apoptotic protein expressions that contribute to caspase cascade activation. Moreover, comparing the drug concentration of BP and BP/LPPC, BP/LPPC was lower in tumor cells that indicated LPPC encapsulation, and thus antitumor efficiency was enhanced. In contrast, BP/LPPC was quicker than BP to induce cell cycle arrest and activate cell apoptosis. The clinical drug 5-FU usually induces strong side effects in patients such as bone marrow suppression; however, BP alone and BP encapsulated by LPPC were less toxic to normal cells. When BP/LPPC was combined with 5-FU, the same concentration of 5-FU led to a decrease in the cell viability of melanoma cells, demonstrating that BP/LPPC had synergistic effects when combined with 5-FU.

Although drawbacks of BP, such as poor water solubility and interaction with serum protein, reduce its scope of medicinal application, nanoparticle drug delivery systems are an alternative method to solve these problems. Several nanoparticles have been developed to improve water solubility and reduce drug interactions with serum albumin; such interactions enhance the stability of the drug [13,14,15,16,17]. The role of LPPC in BP encapsulation was to prevent BP from hydrated or protein-rich environments to stabilize its anticancer activity. The data revealed that during BP/LPPC incubation, LPPC with a positive charge was more easily internalized by tumor cells than control cells. The endocytosis pathway was considered to have been activated by LPPC with a positive charge; therefore, the data indicated that LPPC may have caused the clathrin-mediated, caveolae-mediated, clathrin-independent, caveolae-independent, and micropinocytosis pathways to absorb more BP into tumor cells, thereby causing death. The present study demonstrated that BP and BP/LPPC induced cell cycle arrest and cell apoptosis. The drawbacks of BP were alleviated through LPPC encapsulation to enhance the bioavailability and cytotoxicity of BP to tumor cells.

In conclusion, our study demonstrated that BP possessed cytotoxic activity to melanoma cells and fibroblast sarcoma cells. LPPC carrier encapsulation indeed improves the bioavailability of BP and enhances the toxic activity of BP on tumor cells. Taken together, BP and BP/LPPC inhibited melanoma cells through blocking cell cycle progression and activating cell apoptosis. As a result, BP and BP/LPPC provided a new way to treat melanoma.

## Figures and Tables

**Table 1:** The cytotoxicity (IC_50_) of BP/LPPC in tumor and normal cells.

Cell Line	Tumor Type	BP (a)	BP/LPPC (b)	BP/Lipo	5-FU	Fold (a/b)
Tumor Cell						
B16/F10	mo melanoma	71.47 ± 1.97	12.24 ± 1.22 ^c,d,e^	108.89 ± 2.34	2.66 ± 1.46	5.84
K-balb	mo fibroblast sarcoma	56.49 ± 0.51	15.28 ± 1.94 ^c,d,e^	83.78 ± 2.96	ND	3.70
Normal Cell						
Balb/3T3	mo normal fibroblast	168.8 ± 0.9	50.4 ± 3.6	ND	ND	3.35
SVEC	mo vascular endothelial cell	98.77 ± 0.28	23.36 ± 0.27	ND	9.20 ± 4.66	4.23
MDCK	canine kidney epithelial cell	110.52 ± 1.73	27.05 ± 0.05	ND	>10	4.09

Values revealed mean ± SD (μg/mL) at 24 h. BP/Lipo: BP/Liposome. ND: Non-Detection. SVEC: mouse endothelial cell. MDCK: canis kidney epithelial cell. a: IC_50_ of BP. b: IC_50_ of BP/LPPC. ^c^: *p* < 0.05 BP/LPPC group versus BP group. ^d^: *p* < 0.05 BP/LPPC group versus BP/Lipo group. ^e^: *p* < 0.05 Tumor cells versus normal cells in BP/LPPC group.

**Table 2:** The cell cycle distribution of BP- and BP/LPPC-treated cells.

	**BP (80 µg/mL)**				**BP/LPPC (30 µg/mL)**		
	% G_0_/G_1_	% S	% G_2_/M		% G_0_/G_1_	% S	% G_2_/M
0 h	51.77 ± 1.79	27.75 ± 2.24	20.48 ± 0.51	0 h	50.77 ± 0.62	29.03 ± 0.41	20.20 ± 0.22
6 h	60.38 ± 0.32 *	17.84 ± 0.19 ^#^	21.78 ± 0.38 *	1 h	64.39 ± 0.63 *	19.76 ± 0.41 ^#^	15.85 ± 0.23 ^#^
12 h	62.31 ± 0.59 *	16.15 ± 0.72 ^#^	21.54 ± 0.17 *	3 h	65.66 ± 0.77 *	18.32 ± 0.37 ^#^	16.02 ± 1.12 ^#^
24 h	65.25 ± 1.72 *	17.71 ± 1.69 ^#^	17.04 ± 0.30 ^#^	6 h	67.53 ± 0.30 *	19.37 ± 0.10 ^#^	13.10 ± 0.20 ^#^
48 h	74.80 ± 0.97 *	12.49 ± 0.93 ^#^	12.71 ± 0.19 ^#^	12 h	63.27 ± 1.26 *	23.26 ± 2.14 ^#^	13.48 ± 0.88 ^#^
	**BP (24 h)**				**BP/LPPC (6 h)**		
	% G_0_/G_1_	% S	% G_2_/M		% G_0_/G_1_	% S	% G_2_/M
0 µg/mL	52.05 ± 2.44	27.25 ± 2.93	20.70 ± 0.49	0 µg/mL	52.49 ± 1.82	26.96 ± 2.52	20.55 ± 0.70
40 µg/mL	64.93 ± 0.37 *	17.47 ± 0.30 ^#^	17.60 ± 0.66 ^#^	15 µg/mL	55.23 ± 0.93 *	21.85 ± 0.65 ^#^	22.92 ± 0.39 *
80 µg/mL	66.15 ± 0.52 *	16.94 ± 0.62 ^#^	16.91 ± 0.14 ^#^	30 µg/mL	66.15 ± 0.13 *	21.03 ± 0.37 ^#^	12.82 ± 0.25 ^#^
120 µg/mL	69.81 ± 1.10 *	18.85 ± 2.16 ^#^	11.34 ± 1.38 ^#^	45 µg/mL	71.53 ± 1.51 *	18.11 ± 1.28 ^#^	10.36 ± 0.23 ^#^

Values are mean ± SD (%). ^#^ *p* < 0.05 versus control with significant decrease. * *p* < 0.05 versus control with significant increase.

**Table 3:** The inhibition effect of BP/LPPC cell uptake by endocytosis inhibitor.

	15 min	30 min	45 min	60 min	90 min
No inhibitor	9.70 ± 0.78	12.79 ± 0.35	18.19 ± 3.18	20.70 ± 0.91	22.17 ± 1.57
AHH (13.31 µg/mL)	2.53 ± 0.46 *	3.77 ± 0.72 *	5.50 ± 0.14 *	8.38 ± 0.25 *	8.74 ± 0.14 *
FIII (1 µg/mL)	5.69 ± 1.28 *	6.08 ± 0.46 *	9.71 ± 0.59 *	11.31 ± 0.15 *	12.77 ± 0.06 *
CPZ (10 µg/mL)	2.42 ± 0.25 *	4.66 ± 0.70 *	11.32 ± 0.10 *	11.99 ± 0.11 *	12.42 ± 0.52 *

Values show mean ± SD (μg per 2.5 × 10^5^ cells). * *p* < 0.05 versus control with significant decrease.
